# The impact of obstructive sleep apnea on nonalcoholic fatty liver disease

**DOI:** 10.3389/fendo.2023.1254459

**Published:** 2023-10-02

**Authors:** Haiying Tang, Furong Lv, Peng Zhang, Jia Liu, Jingwei Mao

**Affiliations:** ^1^ Department of Respiratory and Critical Disease, Respiratory Sleep Disorder Center, First Affiliated Hospital of Dalian Medical University, Dalian, Liaoning, China; ^2^ Department of Gastroenterology, First Affiliated Hospital of Dalian Medical University, Dalian, Liaoning, China; ^3^ Department of Medical Information Engineering, Zhongshan College of Dalian Medical University, Dalian, Liaoning, China

**Keywords:** sleep, sleep disorders, intermittent hypoxia, nonalcoholic fatty liver disease, positive airway pressure

## Abstract

Obstructive sleep apnea (OSA) is characterized by episodic sleep state-dependent collapse of the upper airway, with consequent hypoxia, hypercapnia, and arousal from sleep. OSA contributes to multisystem damage; in severe cases, sudden cardiac death might occur. In addition to causing respiratory, cardiovascular and endocrine metabolic diseases, OSA is also closely associated with nonalcoholic fatty liver disease (NAFLD). As the prevalence of OSA and NAFLD increases rapidly, they significantly exert adverse effects on the health of human beings. The authors retrieved relevant documents on OSA and NAFLD from PubMed and Medline. This narrative review elaborates on the current knowledge of OSA and NAFLD, demonstrates the impact of OSA on NAFLD, and clarifies the underlying mechanisms of OSA in the progression of NAFLD. Although there is a lack of sufficient high-quality clinical studies to prove the causal or concomitant relationship between OSA and NAFLD, existing evidence has confirmed the effect of OSA on NAFLD. Elucidating the underlying mechanisms through which OSA impacts NAFLD would hold considerable importance in terms of both prevention and the identification of potential therapeutic targets for NAFLD.

## 1 Introduction

Obstructive sleep apnea (OSA) is characterized by episodic sleep state-dependent collapse of the upper airway, contributing to periodic reductions or cessations in ventilation, with consequent intermittent hypoxia (IH), hypercapnia, arousal from sleep and multisystem damage (1). OSA is increasingly prevalent among children and adolescents, with estimates suggesting a prevalence rate of 1-5% in school-aged children. Furthermore, the prevalence of OSA is notably higher, approximately 4-5 times, in obese adolescents compared to their lean counterparts (2). For adults, statistics indicate that approximately 17%-34% of the middle-aged population meets the diagnostic criteria for OSA (3). As a disease of metabolic stress-induced liver injury, nonalcoholic fatty liver disease (NAFLD) is associated with insulin resistance (IR) and genetic susceptibility, mainly characterized by steatosis and excessive triglyceride accumulation in hepatocytes (4). The global average prevalence of NAFLD is estimated to be approximately 25%; in addition, the prevalence of nonalcoholic steatohepatitis (NASH), a subtype of NAFLD, in male and female NAFLD patients with lifestyle-related diseases was 75%/67% and 53%/54%, respectively (5). In the United States, NAFLD and NASH affect 30% and 5% of the population, respectively (6). In China, with changes in lifestyles over the last 20 years, NAFLD has become the most prevalent liver disorder; data have shown that the prevalence rose sharply from 23.8% in 2001 to 32.9% in 2018 (7). The etiology of liver diseases in China is undergoing a transformation towards NAFLD as a result of the adoption of a westernized lifestyle and the implementation of effective vaccination strategies against hepatitis viruses. Hepatocellular carcinoma (HCC) currently ranks as the sixth most prevalent cause of cancer-related mortality globally, despite the decline in chronic hepatitis infections. This can be attributed to the heightened prevalence of metabolic disorders, including metabolic syndrome, diabetes, obesity, and NAFLD (8). Given the well-known association between obesity and insulin resistance (IR), it can be speculated that there might be associations between OSA severity and IR degree (9). IR is also known to be associated with NAFLD; more importantly, IR is implicated in both NAFLD pathogenesis and progression from steatosis to NASH (10). Therefore, it follows that there should be an association between NAFLD and OSA. Recent studies have provided evidence that OSA and chronic intermittent hypoxia (CIH) are risk factors for NAFLD (11–13). As the prevalence of OSA and NAFLD increases dramatically, these diseases significantly exert adverse effects on the health of human beings, and the medical expenses of these patients are increased. Unfortunately, to date, there have been no effective therapeutic strategies for OSA with NAFLD. In this narrative review, we summarized the influence of OSA on NAFLD and the underlying mechanisms, aiming to explore promising therapeutic targets from the perspective of OSA, thereby preventing the progression of NAFLD combined with OSA.

## 2 Influence of OSA on NAFLD

The direct clinical outcomes of OSA are snoring, increased respiratory efforts, hypercapnia and CIH. For NAFLD patients with OSA, CIH is the critical trigger for oxidative stress increase, reactive oxygen species (ROS) generation, and inflammatory cytokine release, causing systemic inflammation that drives the exacerbation of NAFLD (14). Individuals exposed to CIH could develop hepatocellular inflammation and even liver fibrosis; more importantly, hepatic injury might be exacerbated after challenge with CIH plus other hepatic insults (12). Therefore, CIH presents a key element connecting OSA with NAFLD. OSA exhibits an independent association with obesity, IR, dyslipidemia, and metabolic syndrome (15). Furthermore, NAFLD, specifically NASH, demonstrates a robust association with metabolic syndrome, obesity, and dyslipidemia (16). Emerging evidence indicates that CIH is associated with the development and evolution of NAFLD, independent of metabolic syndrome or its core components. In a single-center study (17), researchers found that patients in the simple snoring and mild, moderate, and severe OSA groups presented 42.86%, 63.5%, 79.4%, and 79.2% hepatic steatosis, respectively, and importantly, the steatosis was significantly different between the simple snoring and the moderate and severe OSA groups. The significance of the apnea hypopnea index (AHI), oxygen reduction index (ODI), lowest oxygen saturation (LaSO2), and mean nocturnal SpO2 (MSaO2) lies in their correlation with NAFLD and OSA, indicating a strong connection between OSA and the progression and intensity of NAFLD (18). In a systematic review and meta-analysis, OSA was found to be correlated with the histological lesions of NAFLD; meanwhile, OSA was also related to alanine aminotransferase (ALT) levels (19). Patients with OSA have increased levels of ALT and aminotransferase (AST) by approximately 13.3% and 4.4%, respectively; in addition, NAFLD patients with OSA have a 2.6-fold higher risk of liver fibrosis (20). More importantly, the severity of OSA was associated with liver fibrosis and NAFLD activity score (NAS), independent of body mass index (BMI), abdominal adiposity, metabolic syndrome, and IR (21). In one cohort study, researchers found that liver fibrosis from F2 to F4 was higher in patients with OSA than in those without OSA, and the prevalence of fibrosis is closely related to LSaO2 attributed to OSA (22). In the IH group, mice were challenged with IH for 12 weeks; consequently, both serum ALT and AST were significantly elevated, and lobular necrosis and portal inflammation were observed in the liver tissue (23). In summary, OSA and, more specifically, CIH were found to have separate associations with the progression of NAFLD, particularly in relation to liver enzyme levels and histological changes.

## 3 Possible mechanism of OSA affecting NAFLD

### 3.1 IR

IR is the key risk factor in the pathophysiology and progression of NAFLD and OSA. The association between OSA, CIH and sleep fragmentation has been proven by an increasing pool of evidence; in addition, OSA is associated with IR and predisposition to type 2 diabetes mellitus (T2DM). Insulin receptor substrate (IRS) proteins are important for the development of NAFLD in the presence of IR, and IR signaling is exclusively mediated by IRS in the liver. Of the four IRS proteins in mammals, IRS1 and IRS2 play key roles in regulating metabolism (24). IRS-1 is one of the critical factors for NAFLD development that is dependent on IR. Surya Prakash Bhatt et al. found that the polymorphism of IRS-1 is an important genetic agent in Asian Indians with OSA and NAFLD (25). However, it is worth noting that additional findings indicate that the disruption of IRS-1 in mice hinders growth, yet it does not lead to the development of diabetes due to the compensatory increase in insulin secretion to counteract the mild insulin resistance. Conversely, the disruption of IRS-2 adversely affects both peripheral insulin signaling and pancreatic β-cell function, resulting in a gradual decline in glucose homeostasis in IRS-2-deficient mice, primarily attributed to liver insulin resistance (26). Furthermore, it was observed that primary hepatocytes lacking IRS-2 were unable to be suppressed in terms of gluconeogenic gene expression by insulin. However, when IRS-2 signaling was reintroduced, these cells regained their response to insulin (27). The liver of hyperglycemic IRS-2(-/-) mice exhibited elevated expression and activity of protein tyrosine phosphatase (PTP)-1B, which impairs insulin signaling mediated by IR/IRS-1 by promoting the association between IR and this phosphatase. In addition, the expression levels of PTP-1B and its association with the IR were found to be increased in the livers of hyperglycemic IRS-2(-/-) mice (28). The implications of these findings suggest that IRS-2 may play a role in mediating IR within the liver. OSA is associated with metabolic abnormalities independently of obesity; OSA or CIH might be a causal factor in the development of IR or T2DM. *In vivo*, mice challenged with IH had disorders of pancreatic β-cell proliferation, cell death and apoptosis and a combination of hyperglycemia (29). CIH exposures mimicking OSA contribute to gut microbiota dysfunction, increase gut permeability, and alter circulating exosome cargo, which lead to adipocyte dysfunction, consequently increasing IR (30). Aoife M Murphy et al. also demonstrated that exposure to CIH induced IR in lean mice by regulating insulin pathways in white adipose tissue (31). Further mechanistic research revealed that CIH-induced IR could be correlated with dysfunction of lipid rafts or caveolae in adipocytes, which was a key event in IR onset and development (32). Sleep fragmentation of OSA contributed to elevated activity of the sympathetic nervous system, hypothalamic–pituitary–adrenal (HPA) axis and oxidative stress reaction, which subsequently led to decreased uptake of insulin-mediated glucose and secretion of insulin, increased levels of ROS, inflammation and pancreatic β-cell apoptosis, consequently resulting in reduced glucose tolerance and IR (14). IH and sleep fragmentation, as hallmarks of OSA, contribute to IR via multiple molecular mechanisms, which are subsequently responsible for the development of NAFLD.

### 3.2 Hypoxia-inducible factors (HIFs)

Hypoxia-inducible factors (HIFs) have been identified as important regulators of inflammation and immunity. During hypoxia, HIFs regulate the transcription of a variety of genes involved in cell processes such as cell survival, inflammation and energy metabolism. Studies have identified an increase in HIF-1α in patients with OSA compared with control subjects (33, 34). HIF-1α is upregulated in the process of NAFLD, where it causes steatosis, suggesting a cause and effect relationship between NAFLD and hypoxia (35). Mice with β-cell-specific HIF-1β knockout were protected from high-fat diet (HFD)-induced diabetes, and similarly, pharmacological HIF-1α inactivation could also prevent or reverse obesity-induced inflammation and IR, which suggested an adverse role of HIF activation in glucose tolerance and IR (36, 37). In another study, researchers found that the levels of fasting blood glucose, glucose tolerance and IR were all improved in mice with hepatocyte-specific deletion of HIF-1α; furthermore, liver changes in IL-1β and TNF-α in both HIF-1α knockout and IH-exposed mice were observed, implying that HIF-1α participated in the process of liver fibrosis in NASH through hepatic inflammation (38). The results indicated that HIF-1α signaling exacerbated metabolic profiles and the progression of NAFLD. HIF-1α is capable of regulating the release of lysyl oxidase (LOX), an enzyme that crosslinks extracellular matrix proteins and promotes liver fibrosis in NASH (39). Patients with liver fibrosis had higher baseline LOX than those without fibrosis; meanwhile, serum LOX was higher in patients with severe OSA than in healthy subjects; however, the level of serum LOX decreased after treatment with continuous positive airway pressure (CPAP) in patients with OSA (40). In addition, mitochondrial dysfunction and oxidative stress mediated by HIF-1 have been identified to be linked with the progression of NAFLD in several studies (41–43) despite one contradictory result that HIF-1 optimized mitochondrial function and protective effects against oxidative stress (38). The rationale might be that mitochondrial dysfunction and oxidative stress may be activated at different time points in NAFLD subjects with OSA, and superimposed HIF-1 attributed to CIH causes variable responses that may fluctuate over time. In addition to HIF-1, HIF-2 also exerts significant influence on physiological and pathophysiological mechanisms within the liver. Activation of HIF-2 resulted in increased inflammation and fibrosis, implying detrimental consequences of IH-induced HIF-2 in the process of NAFLD (44). The continuous activation of HIF-2 in adult individuals leads to the emergence of severe hepatic steatosis, characterized by compromised fatty acid beta-oxidation, reduced expression of lipogenic genes, and heightened lipid storage capacity (45). The activation of HIF-2α in the liver plays a crucial role in regulating liver homeostasis and disease progression. This activation also demonstrates that steatosis, inflammation, and fibrosis are direct consequences initiated by the liver in response to HIF-2α. Furthermore, the activation of HIF-2α serves as a significant mediator in the transition from clinically manageable steatosis to more severe conditions such as steatohepatitis and liver cancer (46). Furthermore, the investigation revealed a notable upregulation of both HIF-2α and CD36 in the liver of individuals with NAFLD. Intriguingly, a significant positive association was observed between hepatic transcript levels of CD36 and erythropoietin (EPO), a gene target dependent on HIF-2α, among NAFLD patients. This finding suggests that HIF-2α plays a crucial role in promoting lipid accumulation in human hepatocytes by enhancing the expression and function of CD36, thereby potentially contributing to the development of hepatosteatosis (47). The aforementioned findings indicate that HIF-2 plays a crucial role in regulating hepatic lipid metabolism, thereby highlighting its potential as a therapeutic target for the management of fatty liver disease. The cellular response to hypoxia is primarily mediated by HIFs and their regulatory enzymes, known as prolyl hydroxylase domain enzymes (PHDs). In normal conditions, HIFs undergo hydroxylation by PHDs, leading to their degradation. However, during hypoxia, the activity of PHDs is suppressed, enabling the accumulation of HIFs and subsequent transactivation of numerous target genes (48). The hepatic vascular and lipid abnormalities were minimized in Phd1^f/+^/(2/3)hKO or Phd(1/2)^f/f^/3^f/+^/hKO mice when a single wild-type Phd1 or Phd3 allele was present. In mice lacking both hepatic PHD2 and PHD3, the liver exhibited normal vascular morphology and lipid deposition. These findings indicate that selective targeting of specific combinations of hepatic PHD isoforms can effectively minimize or prevent liver vascular and lipid defects (49). The treatment of JTZ-951 resulted in an improvement in obesity, inflammation, interstitial fibrosis in white adipose tissue (WAT), and hepatic steatosis through the inhibition of PHD. These findings provide evidence to suggest that targeting PHDs inhibition could serve as a novel therapeutic approach for diseases associated with obesity (50). In summary, recent evidence suggests that HIFs and their regulatory enzymes, specifically PHDs, play a crucial role in the regulation and impact of NAFLD associated with IH or OSA.

### 3.3 Metabolic disturbance of lipids

It is well known that OSA is associated with obesity, dyslipidemia and metabolic syndrome. There is evidence suggesting a positive association between dyslipidemia prevalence and the severity of OSA in patients. Researchers have found that dyslipidemia prevalence increased with OSA prevalence, from non-OSA subjects to mild, moderate and severe patients; more importantly, severe OSA was independently associated with dyslipidemia compared with non-OSA populations (51). The outcomes of OSA, such as high AHI, nocturnal hypoxemia, sleep fragmentation and increased sympathetic activity, may be involved in the process of lipid dysregulation.

An elevation in hepatic triglyceride (TG) levels, induced by IH, was observed, particularly in an obesity mouse model (52). Mechanistically, IH enhances the transport of lipids from adipose tissue to the liver by upregulating sterol regulatory element-binding protein-1 (SREBP-1c), acetyl-CoA carboxylase (ACC), stearoyl-CoA desaturase-1, and fatty acid synthesis (FAS). Additionally, IH promotes the synthesis of cholesterol esters and TG (53). IH leads to excessive production and reduced utilization of free fatty acids (FFAs) by regulating mitochondrial β-oxidation; consequently, an overabundance of FFAs are available for the synthesis of TG and cholesterol, which eventually generates fatty liver (14). In addition, IH has also been shown to be capable of selectively inactivating adipose tissue lipoprotein lipase and reducing very low-density lipoprotein (VLDL) clearance from circulation (54). That is, IH is involved in the onset and progression of NAFLD by breaking the balance between lipid synthesis and catabolism, eventually promoting fat deposition in the liver. In addition to *de novo* lipogenesis (DNL) and FFAs oxidation, the modulation of fatty acid uptake by IH is evident. In patients with NAFLD, there is an increased uptake of circulating lipids, specifically FFAs and lipoproteins, by the liver (55). The accumulation of intracellular lipids induced by hypoxia may be attributed to reduced β-oxidation and enhanced FFAs uptake, rather than an increase in hepatic DNL. Additionally, the regulation of FFAs uptake in various hepatocyte models is influenced by hypoxia and is contingent upon oxygen tension (56). Dysfunction of sympathetic activity, including neurocardiogenic and neurohormonal dysregulation, has been characterized as a consequence of IH in OSA. Hypersympathetic tone participates in the process of lipid metabolic disturbance; in addition, both noradrenaline and cortisol are able to regulate hormone-sensitive lipoprotein and modify the synthesis of high-density lipoprotein (HDL) (57). The overresponse of the sympathetic milieu in patients with OSA might play a critical role in the development of dyslipidemia. Overall, dyslipidemia associated with OSA may serve as a potential mechanism contributing to the onset and progression of NAFLD.

### 3.4 Oxidative stress

Oxidative stress induced by ROS is involved in the process of cell death and liver injury, and it is proposed as a key risk factor in the occurrence and progression of NAFLD (58). In adults with OSA, increased oxidative stress was observed, which improved following treatment with CPAP (59). In the context of pediatric NAFLD, the correlation between oxidative stress and the severity of sleep apnea and hypoxia was found to be significant. This was observed in obese children (60). Moreover, experimental studies involving rats exposed to IH demonstrated a substantial elevation in malondialdehyde (MDA) levels in both the liver and serum. Additionally, IH exposure resulted in histological alterations in the liver, including hepatocyte swelling, necrosis, and infiltration of inflammatory cells, indicating an augmented presence of local and systemic oxidative stress (61). Furthermore, IH exposure led to an upregulation of myeloperoxidase (MPO) expression in the liver, suggesting the activation of oxidative stress within the organism (62). The findings of the experimental research suggested that IH was associated with hepatic injury by inducing elevated levels of oxidative stress and lipid peroxidation. Therefore, current evidence illustrates the role of IH as a trigger for liver oxidative stress, a central mediator associated with the progression of NAFLD in OSA subjects. The following underlying mechanisms may explain the role of oxidative stress in the progression of NAFLD. IR and hyperlipidemia attributed to OSA result in a high level of FFAs with consequent dysfunction of mitochondrial β-oxidation, which leads to ROS generation. Furthermore, obesity associated with OSA, endoplasmic reticulum stress (ERS), and impaired antioxidant production are significant contributors to the excessive production of ROS in the liver. This oxidative injury leads to direct damage to DNA, proteins, and lipids, subsequently triggering the activation of signaling pathways associated with cell death and the impairment of hepatocyte viability and biological function. ROS may also enhance the activation of redox-sensitive transcription factors such as NF-κB and HIF-1, which induce the expression of inflammatory and fibrogenic mediators by Kupffer cells and hepatic stellate cells, thereby aggravating the progression of NAFLD.

### 3.5 Hepatic inflammatory injury

In individuals with morbid obesity, regardless of age, the presence of IR and obesity, IH is significantly linked to elevated levels of systemic and hepatic inflammation, as well as more severe instances of fibrotic or inflammatory liver damage (63). As previously stated, HIFs associated with OSA play a crucial role in the development of hepatic steatosis and inflammation through hypoxia. HIFs govern the transcription of genes involved in inflammation, and their overexpression in hepatocytes, macrophages, and adipocytes stimulates the production of proinflammatory cytokines (64, 65). The activation of HIF in chronic hypoxemic conditions plays a role in the development of hepatic steatosis, inflammation, and fibrosis, ultimately leading to the progression of NAFLD. Conversely, the use of HIF-1α inhibitors has been shown to improve liver fibrosis by downregulating suppressors of cytokine signaling (SOCS) 1 and 3, thereby inhibiting the activation of nuclear factor kappa-B (NF-κB) and phosphorylation of signal transducer and activator of transcription (STAT)3 (66). In addition, hypoxia can also cause liver injury and metabolic inflammation through non-HIF-dependent pathways in NAFLD. The NF-κB signaling cascade enhances the gene transcription of cytokines, promotes the expression of inflammatory factors, such as tumor necrosis factor-alpha (TNF-α) and interleukin (IL-6), as important inflammatory factors affecting hepatic steatosis, are mainly responsible for the induction of neutrophil sequestration in the liver and directly mediate tissue injury (67). CIH promotes early hepatic microcirculation impairment in rats with NAFLD, leading to significant hepatic inflammation and fibrotic changes, despite the presence of HIF expression (68). High induction of inflammatory genes in the liver of IH-exposed mice was observed, suggesting that IH upregulates the inflammatory response in the liver (69). Hypoxia triggers an imbalance in the generation and breakdown of ROS, thereby contributing to the development of oxidative stress. This oxidative stress, in turn, initiates the TLR4/MyD88-dependent pathway, leading to the release of innate cytokines and activation of NF-κB in the liver. Consequently, NF-κB binds to specific target sequences involved in regulating cellular metabolism and the inflammatory response, ultimately promoting the onset of liver steatosis and inflammation (69). In addition, IH can aggravate NAFLD through the RIPK3-dependent necroptosis-modulated Nrf2/NF-κB pathway (70). Therefore, IH mediates the occurrence and development of NAFLD by inducing the oxidative stress response and activating a series of reactions involving NF-κB pathways.

### 3.6 Gut barrier dysfunction

The study revealed that individuals diagnosed with OSA exhibited elevated levels of intestinal permeability and a higher prevalence of NASH, portal hypertension, and significant fibrosis compared to non-OSA patients (71). Furthermore, the researchers observed that the IH associated with OSA is characterized by an altered interaction between the gut and liver, manifested by increased intestinal permeability, endotoxemia, and an overexpression of the endotoxin receptor TLR-4 in hepatocytes, Kupffer cells, and hepatic stellate cells (HSCs). These findings are indicative of key features of NASH and fibrosis in NAFLD. Additionally, researchers have found that IH in OSA is characterized by an altered gut-liver axis, which is characterized by increased intestinal permeability, endotoxemia, and overexpression of the endotoxin receptor TLR-4 by hepatocytes, Kupffer cells and hepatic stellate cells (HSCs), which are key features of NASH and fibrosis in NAFLD. In another study (72), several hepatic and inflammatory parameters were assessed in patients with OSA using two gut barrier markers, intestinal fat acid binding protein (I-FABP) and zonulin in circulation. Researchers found that in patents with OSA, serum I-FABP levels were elevated, and a positive correlation was found between zonulin and aminotransferase levels. The results imply that risk factors for OSA include the condition itself for gut barrier dysfunction; meanwhile, there is a possible connection between intestinal permeability and NAFLD. In terms of mechanism, IH with reoxygenation could contribute to the presence of gut barrier dysfunction in OSA, independent of metabolic disturbances. As a result of the effect of gut wall damage and leaks of macromolecules and microbes into the circulation, hepatic and systemic inflammation are triggered. Emerging evidence suggests that an imbalance in gut bacteria leads to bacterial component translocation and, ultimately, liver injury in the pathogenesis of NAFLD (73–75). The gut microbiota exhibited significant alterations in mice subjected to IH and experiencing systemic inflammation. Metabolic pathway predictions indicate that IH treatment primarily influenced the microbiota’s impact on bile acid and fatty acid metabolism. As a consequence of these findings, dysbiosis of the gut microbiome has been linked with systemic inflammation and metabolism disorders as well as being a mediator for IH (76). IH induces dysbiosis of the gut microbiome, leading to inflammation of the intestinal wall by compromising the integrity of the gut barrier. This, in turn, contributes to intestinal permeability and ultimately promotes liver injury during the progression of NAFLD. In addition to IH, the fragmentation of sleep linked to OSA can alter the intestinal lumen, increasing microbial diversity, inflammatory mediators, and bacterial translocation, i.e., the gut microbiota can be altered by fragmented sleep, leading to hepatic and systemic inflammation and metabolic changes (15). Therefore, reversing gut barrier dysfunction, especially alteration of gut microbiota through prebiotics, probiotics or fecal microbiota transplantation (FMT) combined with CPAP therapy, may open new horizons of treatment to prevent NAFLD.

### 3.7 Sleep deprivation

As a disorder characterized by repetitive episodes of upper airway collapse during sleep, OSA typically leads to arousal, and sleep is often heavily fragmented. Known metabolic risks associated with NAFLD are exacerbated by sleep deprivation attributed to OSA (77, 78). A study by Donghee Kim et al. examined the relationship between sleep duration and quality and indicators of NAFLD among US adults, and the results implied that sleep duration was significantly associated with NAFLD (77). In another clinical study, researchers analyzed the contribution of morningness-eveningness preference to BMI in 2,133 patients with prediabetes and found that patients with prediabetes had more evening preferences that were directly related to higher BMI and indirectly related to insufficient sleep duration (79). In a cross-sectional community-based population study, the odds ratios and 95% confidence intervals for NAFLD prevalence were explored for decreasing sleep duration categories, and the results concluded that short sleep duration was associated with an increased risk of prevalent NAFLD (80). Although sleep deprivation is inversely associated with NAFLD, its mechanisms are not fully understood. Sleep deprivation has been found to potentially increase the likelihood of individuals developing NAFLD through various pathophysiological mechanisms. These mechanisms include disruptions in glucose and lipid homeostasis, as well as proinflammatory stress responses. Maintaining normal liver function requires circadian regulation of the cell cycle, hormone secretion, nutrient absorption, and metabolic flux (81). The pathophysiological ramifications of OSA are linked to the disruption of sleep patterns in a rhythmic manner. Exposure to hypoxic conditions has the potential to modify the expression of circadian genes and the related physiological processes. Of greater significance, prolonged exposure to hypoxic conditions can give rise to disorders frequently connected with the dysregulation of circadian rhythms (82). The deregulation of circadian rhythms has a significant impact on the initiation of NAFLD, as well as its progression to NASH and ultimately HCC (83). The circadian rhythmicity of insulin production and lipogenesis in the liver is thought to be regulated by transcriptional-translational feedback loops controlled by clock genes. Disruption of the circadian clock attributed to sleep deprivation can lead to IR and consequently cause glucose and lipid metabolism disorders, eventually promoting NAFLD-induced hepatocarcinogenesis (84). Circadian genes play a crucial role not only in regulating triglyceride levels and lipid absorption, but also in governing lipid biosynthesis and cellular metabolism. Numerous studies have demonstrated that a considerable number of genes involved in metabolic control are subject to circadian regulation. Consequently, any disruption to this intricate balance may lead to the emergence of severe pathological disorders (85) An increased sympathetic response is linked to sleep deprivation, which can result in low insulin sensitivity and a lower response to glucose (86). There is evidence that sleep deprivation is associated with hormone secretion, including prolonged nocturnal growth hormone release and an early rise in morning adrenaline and noradrenaline, which can have an impact on IR (87). Numerous hormones have been demonstrated to exhibit daily oscillations, with melatonin, cortisol, gonadal steroids, prolactin, thyroid hormone, and growth hormone (GH) being the most extensively studied. Additionally, the nutrient-sensitive hormones, including insulin, leptin, ghrelin, and adiponectin, also display circadian oscillations, which are influenced, to some extent, by environmental cues such as feeding patterns and light-dark cycles (88). Additionally, sleep deprivation can also disturb the rhythm of the inositol-requiring enzyme-1α pathway in the endoplasmic reticulum, leading to inconsistent expression levels of enzymes involved in fatty acid and cholesterol metabolism (89). Sleep-deprived subjects had higher levels of proinflammatory cytokines implicated in NAFLD (90, 91). Consequently, sleep deprivation contributes to the progression of NAFLD by triggering inflammation and oxidative stress. It has been reported that sleep deprivation raises cortisol levels and increases the cortisol awakening response; in addition, sleep deprivation is also known to strengthen the HPA axis, which is thought to exacerbate NAFLD (92–94). Furthermore, sleep deprivation has been shown to aggravate NAFLD by gut barrier dysfunction through inhibiting melatonin secretion, leading to gut oxidative stress and an inflammatory response (95). Consequently, the lack of sufficient sleep is implicated in the pathogenesis of NAFLD via various pathophysiological mechanisms, including compromised glucose and lipid metabolism as well as proinflammatory and stress responses.

### 3.8 Other

OSA has been demonstrated to induce a hypercoagulable state, as evidenced by elevated levels of fibrinogen and other prothrombotic factors, as well as reduced fibrinolytic capacity. The occurrence of various pathophysiological effects of OSA, notably intermittent hypoxia, sympathetic activity, systemic inflammation, and subsequent endothelial dysfunction, may contribute to the development of a hypercoagulable state, platelet activation, and impaired fibrinolytic capacity (96). Patients diagnosed with NAFLD exhibit an elevated susceptibility to cardiovascular events and thromboembolism. Importantly, this heightened risk remains unaffected by the presence of commonly associated metabolic disorders, including diabetes, hyperlipidemia, and obesity. Furthermore, individuals with NAFLD may also face an increased likelihood of developing venous thrombosis in various regions such as the portal, mesenteric, hepatic veins, and lower limbs. The connection between NAFLD and thromboinflammation, characterized by platelet activation and endothelial dysfunction, plays a significant role in the prothrombotic state observed in NAFLD (97). Further research is warranted to explore potential therapeutic targets aimed at preventing vascular disease in patients with NAFLD and improving their clinical outcomes. In patients with OSA and NAFLD, it appears plausible that inhibiting the coagulation system to some extent may potentially slow down the progression of liver disease. This represents a promising objective to pursue in pharmacological research for the treatment of NAFLD.

**Figure 1:**
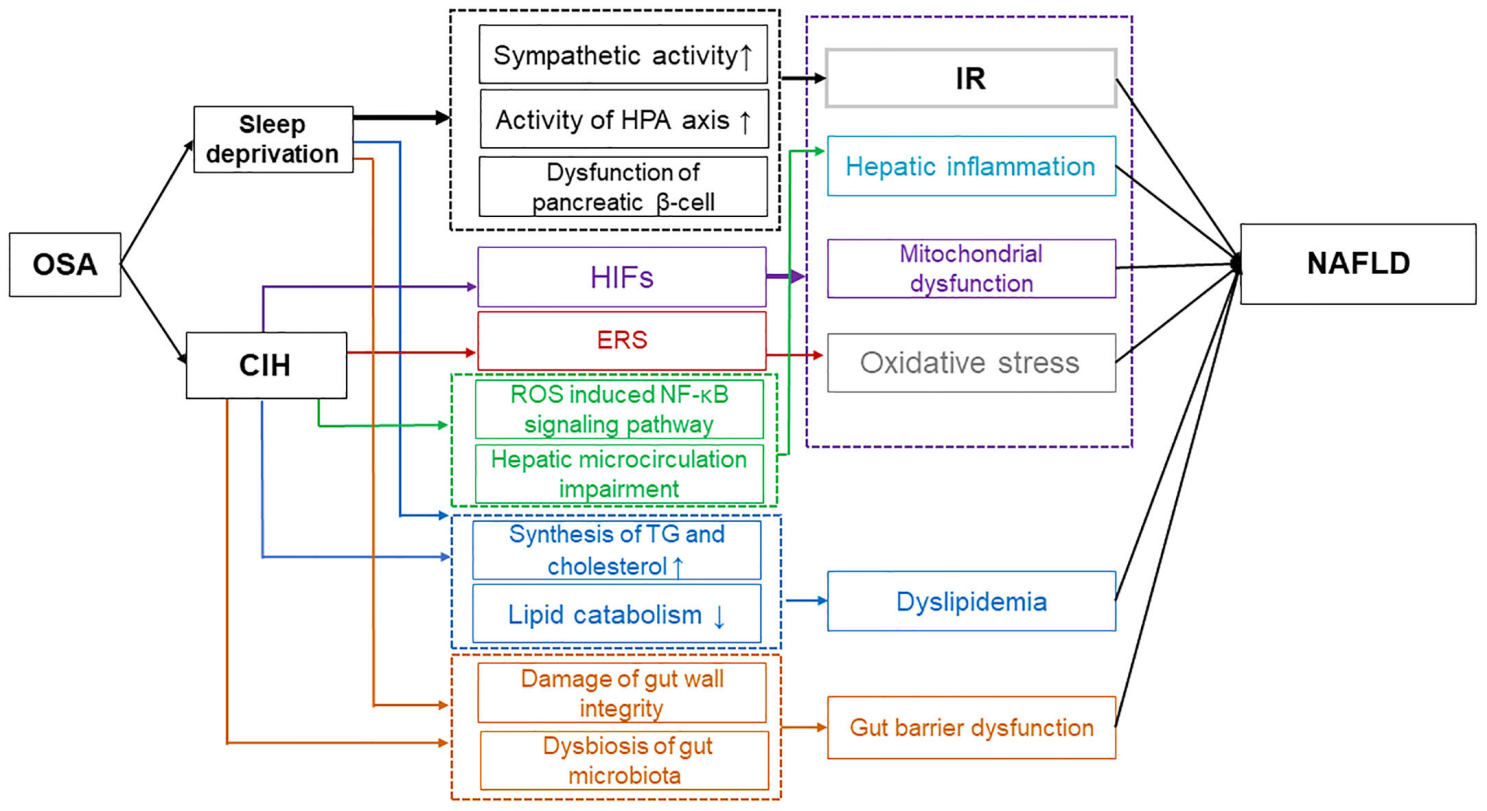
Possible mechanisms of OSA affecting NAFLD. Sleep deprivation caused by OSA has been found to contribute to IR through various mechanisms. These include the increase in sympathetic activity, the strengthening of the HPA axis activity, and the impairment of pancreatic β-cell function. Additionally, sleep deprivation from OSA leads to dyslipidemia by disrupting the balance between lipid synthesis and catabolism, and it also results in gut barrier dysfunction by affecting the integrity of the gut wall and the composition of the microbiota. Ultimately, these factors contribute to the progression of NAFLD. Moreover, CIH contributes to the progression of NAFLD through multiple mechanisms. CIH-induced HIFs exacerbate NAFLD by promoting IR, hepatic inflammation, mitochondrial dysfunction, and oxidative stress. ERS induced by CIH enhances oxidative stress, further impacting NAFLD. Additionally, CIH can induce dyslipidemia and impair gut barrier function, ultimately exacerbating NAFLD. OSA, Obstructive sleep apnea; IR, Insulin resistance; HPA, Hypothalamic-pituitary-adrenal; NAFLD, Non-alcoholic fatty liver disease; CIH, Chronic intermittent hypoxia; HIFs, Hypoxia-inducible factors; ERS, Endoplasmic reticulum stress.

The significance of OSA-induced CIH and sleep deprivation in the advancement of NAFLD is demonstrated in **Figure 1**, as they impact various targets and pathways such as IR, oxidative stress, mitochondrial dysfunction, ERS, hepatic inflammation, dyslipidemia, and gut barrier dysfunction.

## 4 Strategies for ameliorating hypoxemia to improve NAFLD

### 4.1 Positive airway pressure (PAP)

Oxygen deficiency caused by OSA from IH is a critical factor in lipid metabolism dysfunction and accumulation in the liver, leading to steatosis and inflammation of the liver. Hypoxemia correction may therefore improve the severity of NAFLD in patients with OSA. The Clinical Practice Guidelines for the Treatment of Adult Obstructive Sleep Apnea by the American Academy of Sleep Medicine (AASM) recommends that clinicians use PAP, either CPAP or APAP, for on-gonging treatment of OSA in adults (98). It would be expected that effective treatment of OSA with PAP would improve NAFLD if there were a causal relationship between OSA and NAFLD. In an institutional database (2010–2014) based on the study, a statistically significant improvement in biochemical markers and reduction in NAFLD-related fibrosis were observed in patients with OSA who received CPAP treatment for at least three months (99). Serum aminotransferase, a liver injury biomarker, decreased in patients with OSA after three months of treatment with CPAP (100). Noninvasive markers of liver damage are increased in more than 40% of untreated OSA patients (101). In a randomized crossover trial conducted at a single center, the researchers discovered that the recurrence of OSA during CPAP withdrawal leads to elevated levels of FFAs, glucose and cortisol during sleep. These findings were observed alongside increased sympathetic and adrenocortical activation (102). However, there exists a contentious debate surrounding the impact of CPAP treatment on NAFLD in individuals diagnosed with OSA. In a randomized clinical trial, noninvasive markers of hepatic steatosis and fibrosis were not significantly different between therapeutic and subtherapeutic CPAP treatment, i.e., patients with OSA do not benefit from CPAP when they have NAFLD (103). According to the results from a systematic review and meta-analysis, there is no evidence to suggest that CPAP is effective in patients with NASH (104). This could be explained by the fact that CPAP treatment may benefit subjects with OSA regardless of metabolic risk factors for these positive effects to be achieved; however, a sufficiently long therapeutic period is required (perhaps longer than three months). In short, it is possible that CPAP treatment affects NAFLD in OSA subjects by influencing IR, fatty acid dysregulation, oxidative stress, and inflammation factors. For a better understanding of how CPAP affects NAFLD in patients with OSA, more randomized controlled trials (RCTs), particularly those with longer treatment durations, are needed.

### 4.2 Pharmacotherapies

Although PAP is effective in eliminating excessive daytime sleepiness (EDS) in most patients with OSA, a small percentage of patients continue to experience EDS despite using the device. To combat residual EDS, modafinil and its R-isomer, armodafinil, are often prescribed (105). The Food and Drug Administration (FDA) has also recently approved two drugs to treat EDS, solrimfetol, which inhibits the reuptake of dopamine and norepinephrine, and pitolisant, which is an inverse agonist of the histamine H3 receptor (106). Several RCTs conducted on these drugs have demonstrated improvements in subjective and objective tests of EDS and narcolepsy. A 2011 European Medicines Agency decision resulted in the removal of the indication of EDS due to an unfavorable risk-benefit balance in two efficacy trials and accumulated safety information. However, in a systematic review and meta-analysis (107), researchers indicated that there was a significant improvement in the Epworth Sleepiness Score (ESS) with modafinil and armodafinil and a significant advantage over placebo in the Maintenance of Wakefulness Test with modafinil and armodafinil; despite increased adverse events, modafinil and armodafinil had no effect on serious adverse events (hospitalization or death). Solriamfetol’s efficacy in RCTs and nonhepatic renal excretion make it an attractive alternative for individuals taking medications with modafinil-related drug interactions; solriamfetol may also be recommended for patients with persistent residual sleepiness taking modafinil or armodafinil at maximum doses (106). An FDA-approved selective H3-receptor antagonist, pitolisant, was recently approved for treating EDS in narcoleptic patients. According to a double-blind, randomized trial (108), pitolisant was effective in treating EDS compared to placebo and well tolerated compared to modafinil at doses up to 40 mg. There is a low or no potential for abuse with pitolisant, which may be an important potential benefit. Despite their ability to treat OSA to a certain extent, there has been no evidence that they affect NAFLD progression. Recently, a study by Shinkyu Choi et al. revealed that the anti-inflammatory and antifibrotic effects of modafinil in nonalcoholic hepatitis are mediated by KCa2.3- and KCa3.1-mediated pathways rather than by effective treatment of OSA (109).

### 4.3 Other nonpharmacotherapies

#### 4.3.1 Exercise

Obesity serves as a precipitating factor in the development of NAFLD, and its association with OSA is unequivocal (12). That is, among the major modifiable risk factors associated with OSA and NAFLD, obesity is the most important. Weight loss caused by exercise is an effective treatment option, especially for nonsleepy patients with both OSA and metabolic risk (110). Exercise interventions have been shown to be effective in the prevention of NAFLD by a significant body of clinical and preclinical data (111). The severity of OSA and the collapsibility of the upper airways can be reduced in obese individuals with OSA by exercising. It is interesting to note that exercise may improve OSA independently of weight loss. It is likely that fat redistribution, reduced night-time fluid resorption from the legs, and increased pharyngeal muscle strength are the mechanisms responsible for improving sleep quality (112). Regarding the amelioration of OSA severity, it does not seem imperative to attain a specific threshold of weight loss; nevertheless, a positive correlation exists between greater weight loss and more pronounced improvements (113). Exercise might be an effective lifestyle to improve OSA with NAFLD.

#### 4.3.2 Oral appliance therapy, surgery and hypoglossal nerve stimulation

Individuals with mild to moderate OSA can benefit from oral appliances (mandibular repositioning devices) (114). Using these devices increases the volume of the upper airway and reduces the collapsibility of the airways. In patients unable to tolerate PAP therapy, surgical modification of the upper airway may be recommended for select patients. Surgery to treat OSA involves modifying upper airway soft tissue, including the palate, tongue base, and lateral pharyngeal walls. Uvulopalatopharyngoplasty, which entails resection of the uvula and soft palate, is the most researched procedure (112). Furthermore, metabolic and bariatric surgery (MBS) has demonstrated its efficacy and durability as the optimal therapeutic intervention for morbid obesity and its associated ailments. A retrospective study conducted at a single center revealed the effectiveness of MBS in ameliorating sleep apnea, nocturnal hypoxia, liver steatosis, and fibrosis, thereby establishing its crucial role in managing medical conditions in obese patients afflicted with OSA and NAFLD (115). Hypoglossal nerve stimulation during inspiration can also be effective for treating OSA in patients with a BMI of less than 32. In a prospective multicenter single group trial (116), Stimulation Treatment for Apnea Reduction (STAR) trial data indicated that 64% of participants achieved AHI and ODI after 18 months. During the study, quality of life improved, with only two subjects experiencing serious device-related adverse events. However, hypoglossal nerve stimulation is expensive, and approximately 36% of patients do not benefit from it. The above invasive treatments, such as surgical procedures and hypoglossal nerve stimulation, have strict indications, and clinicians need to make treatment plans according to the possible risks and benefits, the patient’s treatment tendency and the accessibility of medical resources. Overall, there is insufficient clinical evidence supporting the improvement of NAFLD through methods other than PAP. Taking into account the varied pathophysiological mechanisms and clinical phenotypes of OSA in addition to its complexity when complicated by NAFLD, a multidisciplinary and individualized treatment strategy should be accepted to correct hypoxia from OSA and achieve the goal of improving NAFLD.

**Table 1** presents a comprehensive summary of the results obtained from recent clinical trials investigating various strategies aimed at mitigating OSA with the objective of enhancing NAFLD outcomes.

**Table 1:** Clinical trials and outcomes of ameliorating OSA to improve NAFLD.

References	Author	Publication year	Study design	Location	Methods	Follow-up length	Average age	No. of Participants	Presented outcomes
(99)	Donghee Kim	2018	Using the Stanford Medicine Research Data Repository (STARR)	USA	CPAP	6 months	57.6 years	351	OSA treatment with CPAP was associated with biochemical improvement and reduction in NAFLD-related fibrosis.
(100)	Li-Da Chen	2018	A single center observational study	China	CPAP	3 months	42.5 years	160	OSA was associated with liver steatosis and serum aminotransferases elevation. After 3 months of CPAP treatment, serum aminotransferases declined.
(101)	Ingrid Jullian-Desayes	2016	Three separate randomized clinical trial	France	CPAP	6-12 weeks	57.0 years	104	Non-invasive markers of liver damage are increased in OSA patients, but they do not improve after 6–12 weeks of effective CPAP treatment.
(102)	Swati Chopra	2017	A single-center randomized crossover trial	USA	Acute CPAP withdrawal vs CPAP	1-4 week washout	50.8 years	31	OSA recurrence during CPAP withdrawal increases FFA and glucose during sleep, associated with sympathetic and adrenocortical activation.
(103)	Susanna SS Ng	2021	A randomized clinical trial	Hong Kong, China	CPAP	6 months	55.0 years	120	CPAP alone did not improve hepatic steatosis and fibrosis.
(115)	Y. X. Zhang	2021	A single-center retrospective study	China	Metabolic and bariatric surgery (MBS)	6 months	33 ± 10 years	127	MBS was effective at improving sleep apnea and nocturnal hypoxia, as well as liver steatosis and fibrosis.

## 5 Conclusions and perspectives

The prevalence of OSA is underestimated, and it is underdiagnosed despite known risk factors and comorbid conditions. While OSA is a disorder primarily of the upper airway during sleep, its pathophysiological impact on other body systems is increasingly recognized. With the increasing prevalence of obesity and weight-related metabolic syndrome, NAFLD has become one of the most prevalent causes of chronic diseases around the world. Although there is a lack of sufficient high-quality clinical studies to prove the causal or concomitant relationship between OSA and NAFLD, existing studies have confirmed the effect of OSA on NAFLD. OSA is associated with the development and evolution of NAFLD, independent of obesity or other shared risk factors. OSA and NAFLD are prevalent disorders associated with major adverse health outcomes.

OSA plays an important role in the progression of NAFLD through multiple pathways, including IR, oxidative stress, mitochondrial dysfunction, ERS, hepatic inflammation, dyslipidemia, and gut barrier dysfunction. There is currently a lack of efficacious pharmaceutical interventions for addressing the co-occurrence of OSA and NAFLD. The hypoxic stress resulting from CIH in OSA plays a pivotal role in the pathogenesis of hepatic lipid metabolism dysfunction and subsequent liver lipid accumulation, ultimately leading to hepatic inflammation and steatosis. Consequently, ameliorating hypoxemia in individuals with OSA has the potential to ameliorate the severity of NAFLD. However, nonpharmacotherapies such as PAP, exercise, oral appliance therapy, surgery and hypoglossal nerve stimulation might be possible approaches for correcting OSA hypoxemia to improve NAFLD. Among these therapeutic approaches, PAP may be beneficial to NAFLD with OSA independent of metabolic risk factors.

The escalating prevalence of obesity has led to a concomitant rise in the occurrence of NAFLD and OSA. Consequently, it can be anticipated that the association between OSA and NAFLD not only represents a burgeoning area of interest within the realm of sleep-breathing disorders but also constitutes a significant research focus within the field of liver diseases. Hence, through the exploration of diverse mechanisms through which OSA impacts NAFLD, basic research holds the potential to unveil novel therapeutic targets for the treatment of NAFLD. In the context of clinical practice, it is recommended that patients with OSA, particularly those who are obese, undergo routine screening using biochemical indicators, liver ultrasound, CT or FibroScan. This screening aims to determine the potential association between OSA and NAFLD, assess the extent of fatty liver, enhance the hypoxic state in these patients, delay the progression of NAFLD, and improve liver function. In summary, a multidisciplinary and personalized treatment approach should be adopted for OSA patients with NAFLD, with the objective of addressing OSA-induced hypoxia and achieving improvements in NAFLD.

## Author contributions

HT: Data curation, Methodology, Writing – original draft. FL: Data curation, Methodology, Writing – original draft. PZ: Writing – review & editing. JL: Writing – review & editing. JM: Conceptualization, Investigation, Supervision, Validation, Writing – review & editing.
